# The *FonSIX6* gene acts as an avirulence effector in the *Fusarium oxysporum* f. sp. *niveum* - watermelon pathosystem

**DOI:** 10.1038/srep28146

**Published:** 2016-06-20

**Authors:** Xiaowei Niu, Xiaoqiang Zhao, Kai-Shu Ling, Amnon Levi, Yuyan Sun, Min Fan

**Affiliations:** 1 Zhejiang Academy of Agricultural Sciences, Institute of Vegetables, Hangzhou, Zhejiang 310021, China; 2 USDA-Agricultural Research Service, U.S. Vegetable Laboratory, Charleston, SC 29414, USA

## Abstract

When infecting a host plant, the fungus *Fusarium oxysporum* secretes several effector proteins into the xylem tissue to promote virulence. However, in a host plant with an innate immune system involving analogous resistance proteins, the fungus effector proteins may trigger resistance, rather than promoting virulence. Identity of the effector genes of *Fusarium oxysporum* f. sp. *niveum* (*Fon*) races that affect watermelon (*Citrullus lanatus*) are currently unknown. In this study, the *SIX6* (secreted in xylem protein 6) gene was identified in *Fon* races 0 and 1 but not in the more virulent *Fon* race 2. Disrupting the *FonSIX6* gene in *Fon* race 1 did not affect the sporulation or growth rate of the fungus but significantly enhanced *Fon* virulence in watermelon, suggesting that the mutant Δ*Fon1SIX6* protein allowed evasion of R protein-mediated host resistance. Complementation of the wild-type race 2 (which lacks *FonSIX6*) with *FonSIX6* reduced its virulence. These results provide evidence supporting the hypothesis that *FonSIX6* is an avirulence gene. The identification of FonSix6 as an avirulence factor may be a first step in understanding the mechanisms of *Fon* virulence and resistance in watermelon and further elucidating the role of Six6 in *Fusarium*-plant interactions.

Watermelon [*Citrullus lanatus* (Tunb.) Matsum. & Nakai] is an important cucurbit crop accounting for 7% of the agricultural land area devoted to vegetable production worldwide. The total annual production of watermelon is approximately 90 million tons, making it among the top five most consumed fresh fruits (http://faostat.fao.org). Fusarium wilt, caused by the soil-borne fungus *Fusarium oxysporum* f. sp. *niveum* (*Fon*), is a major disease of watermelon throughout the world, with a large adverse impact on watermelon yield and quality^1^.

There are three common physiological races (0, 1 and 2) of this pathogen, classified according to their reactions with differential watermelon genotypes (Table 1)^2^,^3^,^4^. Race 0 is pathogenic only in watermelon cultivars with no resistance genes. Race 1 is the predominant race throughout commercial watermelon regions worldwide, and several watermelon cultivars, such as cv. Calhoun Gray, are resistant to this race. Race 2 is highly aggressive to all current commercial watermelon cultivars and hybrids. Race 3, the most virulent race of *Fon* described to date, was shown to cause over 90% wilt on PI296341-*FR*, whereas no disease was caused by a race 2 isolate^5^.

The co-evolution of plants and microorganisms involves complex mechanisms of attack and defence, implicating the innate immune system of plants and virulence factors of pathogens^6^. The first layer of plant defence, known as basal immunity, is based on the recognition of conserved microbial molecules but can be suppressed by microbial virulence factors known as “effectors”. Plants respond to this suppression by employing a second layer of defence, resistance (*R*) gene-based immunity, which relies on the recognition of effectors^7^. Finally, the pathogen evolves further and escapes detection by the *R* gene product by eliminating the detected virulence factor or by suppressing the defence induced by *R* gene products^8^. Effectors may be defined as pathogen proteins and small molecules that alter host-cell structure and function. These alterations either facilitate infection (virulence factors and toxins) or trigger defence responses (avirulence factors and elicitors), or both^9^.

The secreted effector proteins of *F. oxysporum* f. sp. *lycopersici* (*Fol*) infecting tomato have been identified through proteomic analysis of xylem sap from tomato plants infected with *Fol.* These proteins have been designated the Six (secreted in xylem) proteins and include Six1 to Six7^10^,^11^. Several functions of the Six proteins have been identified thus far. Avr3 (Six1) is required for *I-3*-mediated resistance^12^, and Avr1 (Six4) is required for *I*-mediated resistance^13^. Additionally, both proteins have functions other than triggering avirulence: Avr3 is required for full virulence^14^, whereas Avr1 suppresses *I-2*- and *I-3*-mediated disease resistance^13^. Subsequently, Avr2 (Six3) shows both activities: it is required for full virulence in susceptible tomato host plants while triggering resistance in plants carrying the resistance gene *I-2*^15^. Six5 is required for full virulence in susceptible plants, and knockout of this gene can breach *I-2*-mediated disease resistance. Avr2 and Six5 interact in yeast two-hybrid assays as well as *in planta*. The *AVR2-SIX5* gene pair is required to activate *I-2*-mediated immunity in tomato^16^, while Six6 is a true effector that enhances virulence and simultaneously suppresses *I-2*-mediated cell death^17^. Screening of effector proteins indicated that the three *AVR* gene sequences (*AVR1, AVR2*, and *AVR3*) and the *SIX5* gene sequence are not present in *Fon* races, while the *SIX6* gene homologue is present^11^, although its biological function has not been determined^18^.

In this study, we identified and analysed the biological function of the *SIX6* gene in *Fon* (*FonSIX6*) and demonstrated that *FonSIX6* is an *AVR* gene playing a key role in the *Fon*-watermelon pathosystem.

## Results

### Cloning and analysis of *FonSIX6* and flanking sequences

Using the genome sequence of *Fol* (http://www.broadinstitute.org/) as a reference for constructing PCR primers, we cloned a *SIX6* gene of *Fon.* Here, we used primers that annealed immediately outside the *FolSIX6* gene ORF^11^. The resultant gene was designated *FonSIX6*. Genome searches using the *FonSIX6* ORF sequence as a query showed high sequence homology of *SIX6* with *F. oxysporum* f. sp. *melonis* (*Fom*, 100%) and *Fol* (94.91%) (Supplementary Fig. S1). To obtain additional information about *FonSIX6*, the *Fol* genome was used as a reference for designing specific PCR primers for the flanking sequence. However, no PCR fragments were amplified, suggesting that the *Fol* and *Fon* genomic sequences are different. Finally, the 1974 bp upstream sequence (directly adjacent to the start codon, −1974 bp) and the 453 bp downstream (directly 3′ to the stop codon, +453 bp) of the *FonSIX6* open reading frame were cloned via chromosome walking (Supplementary Fig. S2).

### Analysis of the conserved *FonSIX6* homologue sequence in a watermelon-infecting strain

**Figure 1:**
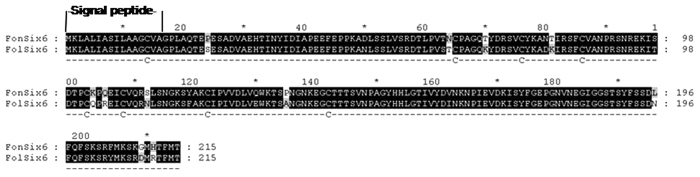
Amino acid alignment of putative Six6 from FonSix6 (*Fusarium oxysporum* f. sp. *niveum*) with FolSix6 (*F. oxysporum* f. sp. *lycopersici*). The signal peptide sequence predicted by the Signal P program (http://www.cbs.dtu.dk/services/SignalP/) is depicted, and the eight cysteine residues are marked below the sequence. FonSix6: the putative amino acid sequence from *Fusarium oxysporum* f. sp. *niveum*, the nucleotide sequence cloned from wild race 1; Fol*Six*6: *Fusarium oxysporum* f. sp. *lycopersici* (ACY39286.1).

**Figure 2:**
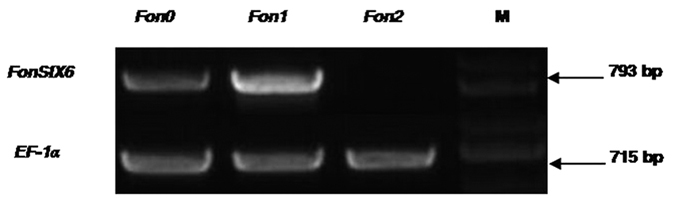
The presence of *SIX6* in *F. oxysporum* f. sp. *niveum*. *Fon* 0, *Fon* 1, *Fon* 2: *F. oxysporum* f. sp. *niveum* wild race 0, race 1 and race 2. *FonSIX6*: the *SIX6* gene from *F. oxysporum* f. sp. *niveum*; *EF-1α*: elongation factor of *F. oxysporum* f. sp. *niveum*. M: marker lane (DL 2000, Takara).

The deduced FonSix6 protein consists of 215 amino acids and contains 8 cysteine residues (Fig. 1). This intronless gene is predicted to encode a 21.85 kDa mature protein (after cleavage of the predicted N-terminal signal peptide) (http://web.expasy.org/compute_pi/). Amino acid sequence comparisons between FonSix6 and FolSix6 (NCBI GenBank:ACN69116.1) showed 90.23% identity, with minor differences. Using the SignalP 4.0 Server, the FonSix6 protein was predicted to contain a signal peptide consisting of 16 amino acids at its N-terminus (http://www.cbs.dtu.dk/services/SignalP) (Fig. 1). Among the three physiological races (0, 1 and 2) of the *Fon* pathogen, the *FonSIX6* gene sequence exists in the genomes of *Fon* races 0 and 1 but not in the more aggressive race 2 (Fig. 2).

### Impact of *FonSIX6* disruption on fungal development

To determine whether *FonSIX6* affects fungal growth and microsclerotia production in *Fon*, the growth patterns of the gene disruption mutant Δ*FonSIX6* on potato dextrose agar (PDA), minimal medium (MM) and complete medium (CM) media were compared with those of the wild-type *Fon* strain and the gene-complemented Δ*Fon1SIX6* + *SIX6* and *Fon2* + *SIX6* strains.

**Figure 3:**
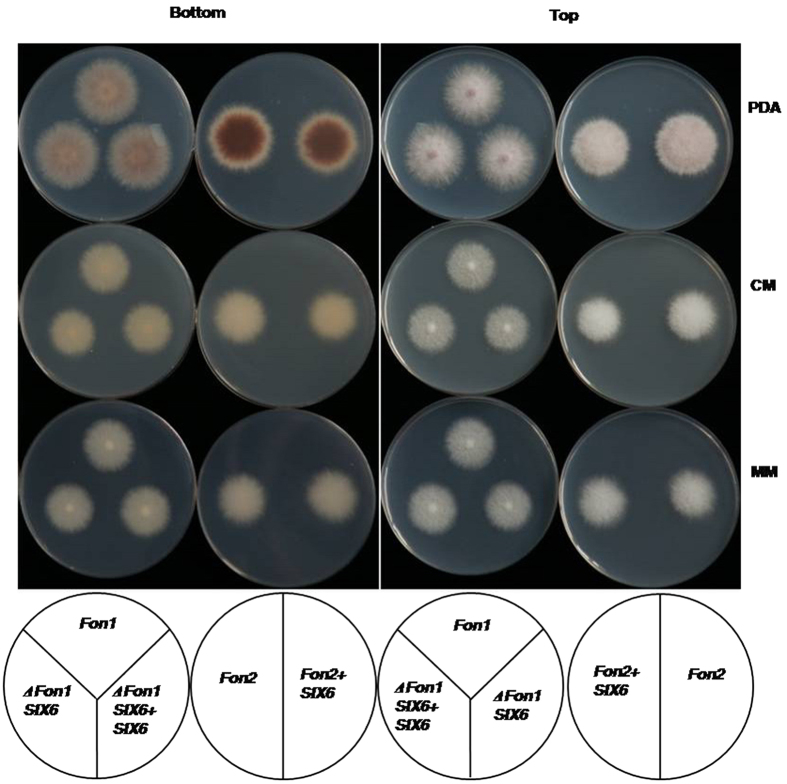
Colony growth of deletion and complementation mutants compared with wild-type *Fon* on PDA, CM, and MM. *Fon1*: *Fusarium oxysporum* f. sp. *niveum* wild race 1; *Fon2*: *Fusarium oxysporum* f. sp. *niveum* wild race 2; Δ*Fon1SIX6*: race 1 with *SIX6* disrupted by gene replacement; Δ*Fon1SIX6* + *SIX6*: Δ*Fon1SIX6* transformed with *SIX6*; *Fon2* + *SIX6*: race 2 transformed with *SIX6*; PDA: potato dextrose agar medium; MM: minimal medium; CM: complete medium. The photographs were taken from the top and bottom of the plates 4 days after incubation.

During growth on PDA, the *Fon* mycelium produced red-brown pigments, whereas no red-brown colour was observed on MM or CM medium. Measurement of colony diameter on PDA, MM, or CM medium during the first 4 days of culture indicated that the radial growth of the *FonSIX6*-disrupted and complemented strains did not differ from that of the wild-type strain (Fig. 3). The morphology and quantity of spores also did not differ substantially, as observed under a microscope. These observations indicate that the *FonSIX6* gene is not essential for *Fon* growth and development.

### *FonSIX6* is expressed 3 days after Fon infection

**Figure 4:**
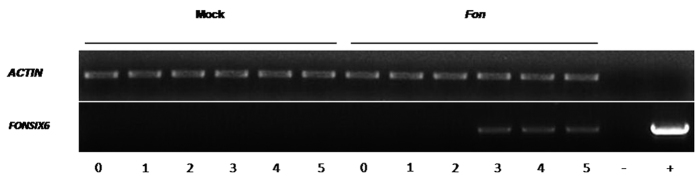
*FonSIX6* is expressed during the early stages of infection. Reverse-transcriptase polymerase chain reaction analysis of watermelon actin (*ACTIN*) or *FonSIX6* expression using RNA isolated from the roots of watermelon seedlings, which were either mock or *Fusarium oxysporum* f. sp. *niveum* inoculated and harvested on the 1^st^, 2^nd^, 3^rd^, 4^th^ and 5^th^ day post-inoculation (DPI). Water was included as a negative control (−), while genomic DNA from *Fusarium oxysporum* f. sp. *niveum* (+) was used as a positive control.

To determine whether *FonSIX6* was expressed during earlier stages of infection, RNA was collected from *Fon*-infected watermelon roots on the 1^st^, 2^nd^, 3^rd^, 4^th^ and 5^th^ day post-inoculation (DPI). The expression of *FonSIX6* was monitored using reverse-transcriptase polymerase chain reaction (RT-PCR). *FonSIX6* transcripts could be detected on the 3^rd^ through the 5^th^ DPI in infected plants, whereas the mock-inoculated controls did not produce this transcript (Fig. 4).

### Watermelon inoculation with wild-type and transformants

**Figure 5:**
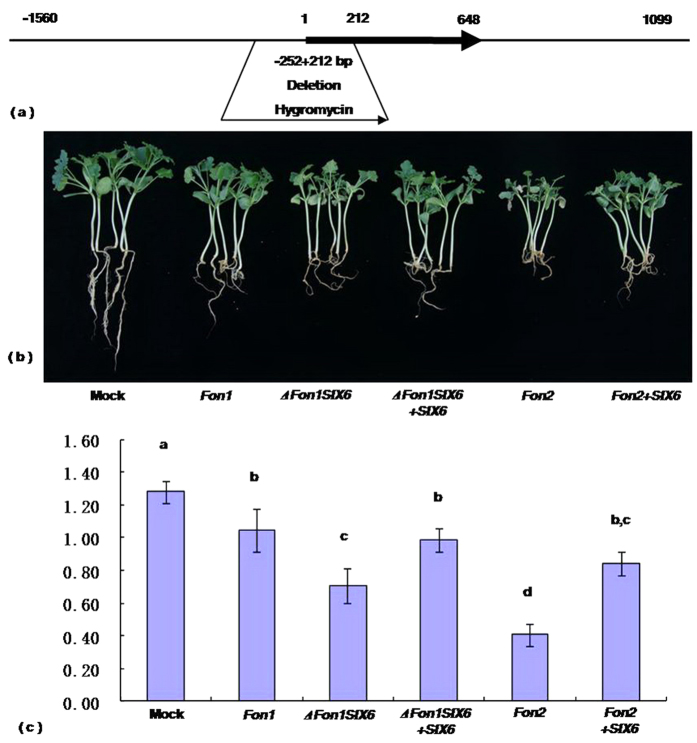
Watermelon plants were inoculated with *Fon 1*, Δ*Fon1SIX6*, Δ*Fon1SIX6* + *SIX6*, *Fon 2* and *Fon 2* + *SIX6* and the development of disease symptoms was assessed. Seedlings (cv. Calhoun Gray, resistance to *Fon 1*) at the first true leaf stage were inoculated with a fungal spore suspension, and disease symptoms were scored after 15 days. (**a**) The Δ*FonSIX6* mutants were generated using *Agrobacterium*-mediated targeted disruption of the *SIX6* gene. (**b**) Representative plants are shown 15 days post-inoculation. (**c**) Quantification of disease assays by weight. The outcomes of the disease assays depicted in (**b**) were quantified based on the average plant weight from each inoculation. Error bars indicate standard deviation and letters indicate values that are significantly different from each other (P < 0.01, all pairs Student’s *t*-test). All of the assays were repeated at least three times.

To determine the role of *FonSIX6* in the infection of watermelon, *FonSIX6* knockout mutants (Δ*Fon1SIX6*) were generated in *Fon* race 1 by replacing *FonSIX6* with a hygromycin resistance cassette (Fig. 5a). Then, the virulence of the Δ*FonSIX6* strain was assessed by inoculating watermelon seedlings (cv. Calhoun Gray, resistant to *Fon* 1). To our surprise, the severity of disease symptoms in watermelon plants inoculated with Δ*Fon1SIX6* transformants were significantly enhanced (severe, Fig. 5b,c). Reintroduction of the *FonSIX6* gene into Δ*Fon1SIX6* led to disease symptoms similar to those associated with wild-type *Fon* 1 (mild, Fig. 5b,c). On the other hand, when symptom expression was compared in watermelon plants inoculated with *Fon2* + *SIX6* transformants and the more aggressive wild-type *Fon* 2 (lack of *SIX6*), the severity of disease symptoms was significantly reduced in plants inoculated with *Fon2* + *SIX6* transformants compared with those inoculated with wild-type *Fon* 2 (Fig. 5b,c). These results indicated that the mutant Δ*FonSIX6* protein allowed evasion of host resistance mediated by the R protein. Therefore, *FonSIX6* is an avirulence factor. Complementation of wild-type race 2 with the *FonSIX6* gene reduced race 2 virulence, further confirming that *FonSIX6* is an *AVR* gene.

## Discussion

The vascular pathogen *F. oxysporum* is an asexual fungus with a broad host range that causes wilt and root diseases in many economically important crop plants, including watermelon^19^. In *Fol*, 14 ‘Secreted in xylem’ (Six) proteins (Six1~14) have been identified from *Fol*-infected tomato plants^10^,^20^. Of the Six proteins, Six6 contributes to virulence and suppresses *I-2*-mediated cell death. Although a Six6 homologue sequence has been identified in *Fon* isolates, its functional has not been characterized^17^,^18^. In this report, we describe the identification and functional analysis of the *SIX6* gene of *Fon*.

Earlier studies showed that the *FonSIX6* gene was present in the *forma specialis niveum* in isolates 546 and 704 but not in isolates 703, 705, CBS 187.60, CBS 418.90, and CBS 419.90^11^,^18^. Three generally accepted physiological races (0, 1 and 2) of the *Fon* pathogen have been identified to date, according to their effects in differential watermelon genotypes^2^,^3^,^4^. This study indicates the possibility that the *FonSIX6* gene is present in races 0 and 1 but not in race 2. Based on these results, we speculate that isolates 703, 705, CBS 187.60, CBS 418.90, and CBS 419.90 likely belong to race 2 while the other isolates are likely from races 0 or 1.

Although the pathogenicity of *Fon* is gradually increased in races 0, 1 and 2, there is growing speculation that the distinction between race 0 and race 1 may be more quantitative than qualitative. Consequently, races 0 and 1 might be strains of race 1 with varying aggressiveness. On the other hand, race 2 is highly aggressive to all current commercial watermelon cultivars and hybrids and is clearly a distinct race^2^. Here, the association of the *FonSIX6* gene with the pathogenicity of different *Fon* races may provide a potential cultivar-specific pathogenicity marker that is useful for defining host targets and evolutionary bottlenecks that control the *Fon*-watermelon pathosystem.

In *Fol*, most *SIX* genes are located in the same lineage-specific (LS) genomic region- chromosome (chromosome 14), also known as the pathogenicity chromosome, and are associated with chromosomal sub-regions enriched for DNA transposons. The LS genome regions could have been acquired through horizontal transfer from another species, leading to the hypothesis that horizontal chromosome transfer in *F. oxysporum* can generate new pathogenic lineages^21^,^22^. *FolSIX6* is located on a supernumerary chromosome 14, an LS chromosome^21^. Searches carried out using the *FonSIX6* ORF sequence as a query showed strong sequence identity to *SIX6* of *Fom* (100%) and *Fol* (94.91%) (Supplementary Fig. S1). Comparison of the *FonSIX6* ORF and its flanking sequences between the *Fom* and *Fol* genomes in the −609 ~ +157 and −433 ~ +157 regions showed nucleotide sequence identities of 99.58% and 96.29%, respectively (Supplementary Fig. S2). The genome of *F. oxysporum* 4287 (FO2) has been sequenced and is available (http://www.broadinstitute.org/). BLAST searches of this genome sequence database using the *FonSIX6* sequence segment (−1974 ~ +453) as a query showed the presence of high sequence identity (90 ~ 100%) in 5 distinct segments. Segment 1 (−1974 ~ −686) is located in supercontig 37 of Chr15 (91.47% nucleotide sequence identity). Segments 2 ~ 4 are located on supercontig 22 of Chr14 at different positions with various nucleotide sequence identities, with segment 2 (−685 ~ −654) showing 100% identity, segment 3 (−653 ~ −434) showing 95% identity and the segment 4 (−433 ~ +157) showing 96.29% identity, while segment 5 (+158 ~ +453), located on nonpositional scaffolds, exhibited 98.31% nucleotide sequence identity. These analyses suggested that the −433 ~ +157 sequence segment identified in *Fon* may contain the full gene sequences that are necessary to complete the function of *FonSIX6*.

Here, we generated *FonSIX6* gene knockout mutants in race 1, evaluated the complementation of the knockout mutants and wild-type race 2 with the *FonSIX6* gene, and observed that the disruption of *FonSIX6* did not affect the growth rate or fungal sporulation. These results demonstrate that *FonSIX6* is not absolutely necessary for *Fon* growth and development. Therefore, the change in the virulence of Δ*FonSIX6* is not associated with fungal growth or development and is instead due to the effector’s key role in the *Fon*-watermelon pathosystem.

In comparison with the watermelon plants inoculated with wild-type *Fon*, the severity of the disease symptoms of the watermelon plants inoculated with the Δ*Fon1SIX6* transformants was significantly enhanced. These results suggest that the mutant Δ*Fon1SIX6* protein allowed evasion of R protein-mediated host resistance. On the other hand, complementation of wild-type *Fon* 2 (lacking *FonSIX6*) with the *FonSIX6* gene reduced its virulence. Taken together, these results indicate that *FonSIX6* is an *AVR* gene. Loss of function of an *AVR* gene (*FonSIX6*) in race 2 allowed the pathogen to avoid the induction of resistance in a watermelon cultivar. Thus, the pathogen gained pathogenicity in that cultivar, and a new pathogenic race (race 2) emerged. The three known races (1, 2 and 3) carry *AVR* genes in different combinations in *Fol*. *Fol* race 2 emerged from race 1 by losing *AVR1* and thereby allowed evasion of host resistance mediated by *I* (the resistance gene corresponding to *AVR1*). Race 3 emerged when race 2 sustained a point mutation in *AVR2*, allowing it to evade *I-2*-mediated resistance of the host^15^,^23^. The results of the present study indicate that *Fon* race 2 may have emerged from race 1 owing to loss of the entire *FonSIX6* gene sequence or may have resulted from a mutation that impaired the function of the *FonSIX6* gene, evading mediated host resistance. Additional studies are needed to further determine the differences between *Fon* races 1 and 2.

## Methods

### Alignment

DNA sequence and protein alignments were performed using the computer programs ClustalW and DNAMAN. The genome of *Fol* (http://www.broadinstitute.org) was used as a reference sequence for constructing PCR primers to clone the homologous *SIX6* gene sequence of *Fon.*

### *Fon* races and mutant lines used in this study

The following *Fon* strains were used: *Fon* 0 (race 0), *Fon* 1 (race 1), and *Fon* 2 (race 2) (a kind gift from the National Engineering Research Center for Vegetables, Beijing, China). Δ*Fon1SIX6* was race 1 with *SIX6* disrupted by gene replacement. Δ*Fon1SIX6* + *SIX6* was Δ*Fon1SIX6* transformed with *SIX6*. *Fon2* + *SIX6* was race 2 transformed with *SIX6*.

### *FonSIX6* disruption and complementation constructs

The *FonSIX6* flanking sequence was cloned using the Genome Walker Universal kit (Clontech). The *FonSIX6* disruption construct was generated via PCR amplification of *FonSIX6* upstream and downstream sequences (with partial *FonSIX6* sequences) for homologous recombination, followed by insertion in front of and behind the hygromycin resistance gene in the vector pDHt2^24^. An upstream fragment, from 1560 to 252 bp upstream of the start codon, was cloned into pDHt2 between the *Eco*R I (5′>CCGGAATTCACGCTCTGTATGCCTGCTC<3′) and *Sac* I (5′>CGAGCTCGTCGGTGAATGGTATGTTGTTT<3′) sites, and a downstream fragment, from 212 bp after the start codon to 1099 bp downstream of the stop codon, was cloned into pDHt2 between the *Sac* I (5′>CGAGCTCTGACCGCTCCGTCTGCTA<3′) and *Xba* I (5′>TCTCCTCTAGAATCGACGCGGCTGTAAGGAT<3′) sites (Fig. 5a). Transformants were selected on hygromycin B and confirmed by PCR (Supplementary Fig. S3).

To generate a *FonSIX6* complementation construct, a fragment of 2659 bp containing the *FonSIX6* open reading frame, 1560 bp of upstream sequence and 451 bp of downstream sequence was amplified via PCR using primers with *Eco*R I and *Xba* I linkers (underlined) (5′>CCGGAATTCACGCTCTGTATGCCTGCTC<3′ and 5′>TCTCCTCTAGAATCGACGCGGCTGTAAGGAT<3′). This fragment was cloned into pCOM^24^. Transformants were selected on geneticin and confirmed through PCR (Supplementary Figs S4 and S5). Transformation of the constructs into *Fon* was carried out using *Agrobacterium* as described previously^25^.

### Plant material and fungal strains

The following watermelon differentials were used: cv. Sugar Baby and cv. Calhoun Gray. *Fon* race 0 causes wilt in cv. Sugar Baby; *Fon* race 1 causes wilt in cv. Sugar Baby and cv. Charleston Gray but not in cv. Calhoun Gray; and *Fon* race 2 causes wilt in all of the differential cultivars but not in PI296341-FR^4^.

### Pathogenicity assay

Each *Fon* isolate was cultured on potato sucrose broth (PSB) for 5 days at 25 °C at 120 rpm, and conidial suspensions (1.0 × 10^6^ conidia ml^−1^) were prepared.

Seeds of each cultivar were sown in vermiculite in plastic pots (6 by 6 by 5 cm, 32 cells tray^−1^) and grown in a greenhouse set at 24–30 °C on top of a heat pad (30 °C). The standard root dip method was used to inoculate watermelon seedlings. At the first true leaf stage, the seedlings were dipped in a conidial suspension (1.0 × 10^6^ conidia ml^−1^) for 5 min and replanted to a vermiculite tray. Disease was scored at 15 days post-inoculation. The disease assay results were quantified based on the average plant weight and the typical disease symptoms of yellowing, stunting and wilting. Because the inoculation methods involving direct dipping or root cutting yielded a similar disease incidence and symptom severity, the data obtained using the two methods were combined for analysis. All of the tests were repeated at least three times.

### RNA isolation and RT-PCR

For *FonSIX6* gene expression analysis, RT-PCR experiments were performed using tissue harvested from *Fon*1-infected roots of watermelon cv. Sugar baby. The root samples were ground in liquid nitrogen. Then, total RNA was extracted with the RNAiso plus reagent (Takara), and DNA was removed with recombinant DNase I (Takara). cDNA was subsequently synthesized using the PrimeScript 1st Strand cDNA Synthesis Kit (Takara). The primer combinations w-actinF/w-actinR (W-actinF: 5′>AATGTGCCTGCTATGTATGTCG<3′; W-actinR: 5′>GATGGAGTTGTAGGTAGTTTCG<3′) and Fon*SIX*6F/Fon*SIX*6R (Fon*SIX*6F:5′>CGCTCTTATCGCATCAATCT<3′; Fon*SIX*6R:5′>GGGTTGACTGAGGTCGTGGT<3′) were used to amplify the watermelon actin gene and *FonSIX6*.

### Vegetative growth, conidiation and microsclerotia formation assays

For each sample, a 0.5 μL drop of a conidial suspension (1.0 × 10^6^ conidia ml^−1^) was inoculated onto the centre of a 90-mm Petri dish containing potato dextrose agar (PDA), minimal medium (MM), and complete medium (CM)^26^ and cultured at 25 °C. The colony diameter and morphology of the vegetative mycelia were examined at 4 days after inoculation. To estimate conidial production, discs of 7 mm in diameter obtained from the edge of a 10 day-old fungal colony on PDA medium were suspended in 1 mL sterilized water, then subjected to shaking at 150 rpm for 10 min. A 100 mL drop of the conidial suspension was subsequently placed onto a haemocytometer, and the spores were counted under a microscope. All of the tests were repeated at least three times.

## Additional Information

**Accession codes:** Sequence data for *FonSIX6* with flanking sequences from this paper have been deposited with the EMBL/DDBJ/GenBank data libraries under accession no. LT160066.1.

**How to cite this article**: Niu, X. *et al*. The *FonSIX6* gene acts as an avirulence effector in the *Fusarium oxysporum* f. sp. *niveum* - watermelon pathosystem. *Sci. Rep.* **6**, 28146; doi: 10.1038/srep28146 (2016).

## Supplementary Material

Supplementary Information

## Figures and Tables

**Table 1:** Watermelon genotypes used to differentiate races of *Fusarium oxysporum* f. sp. *niveum*.

Cultivar or genotype	Disease response to:^*^		
	Race 0	Race 1	Race 2
Sugar Baby	S	S	S
Charleston Gray	R	S	S
Calhoun Gray	R	R	S
PI 296341-FR	R	R	R

^*^S = susceptible. R = resistant.
