# Supplementary material for: The FonSIX6 gene acts as an avirulence effector in the Fusarium oxysporum f. sp. niveum - watermelon pathosystem
Source: Sci Rep. 2016 Jun 20;6:28146. doi: 10.1038/srep28146 (PMC4913299; doi:10.1038/srep28146)
Supplement: Supplementary Information [file srep28146-s1.pdf]

The *FonSIX6* gene acts as an avirulence effector in the *Fusarium oxysporum* f. sp. *niveum* - watermelon pathosystem

Xiaowei Niu[1], Xiaoqiang Zhao[1], Kai-Shu Ling[2], Amnon Levi[2], Yuyan Sun[1], Min Fan*[1]

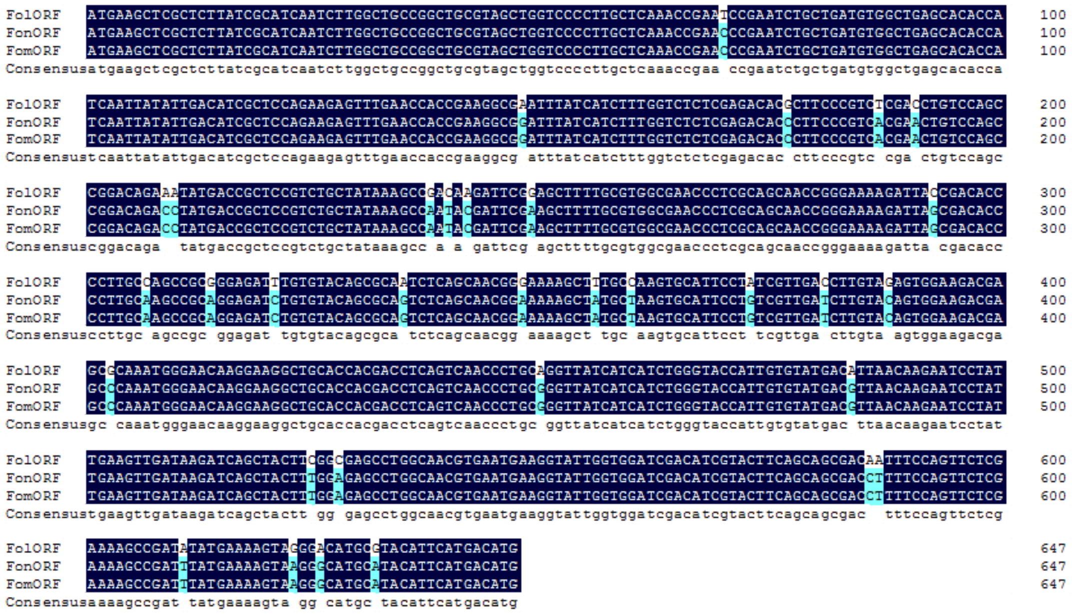

Supplementary Fig. S1 Nucleotide sequence alignment of *SIX6* ORF

FonORF, The sequence of *FonSIX6* was cloned from *Fusarium oxysporum* f. sp. *niveum* race1; The ORF sequences of *FolSIX6* and *FomSIX6* are derived from the genome sequences of *F. oxysporum* f. sp. *lycopersici* and *F. oxysporum* f. sp. *melonis* (http://www.broad.mit.edu).

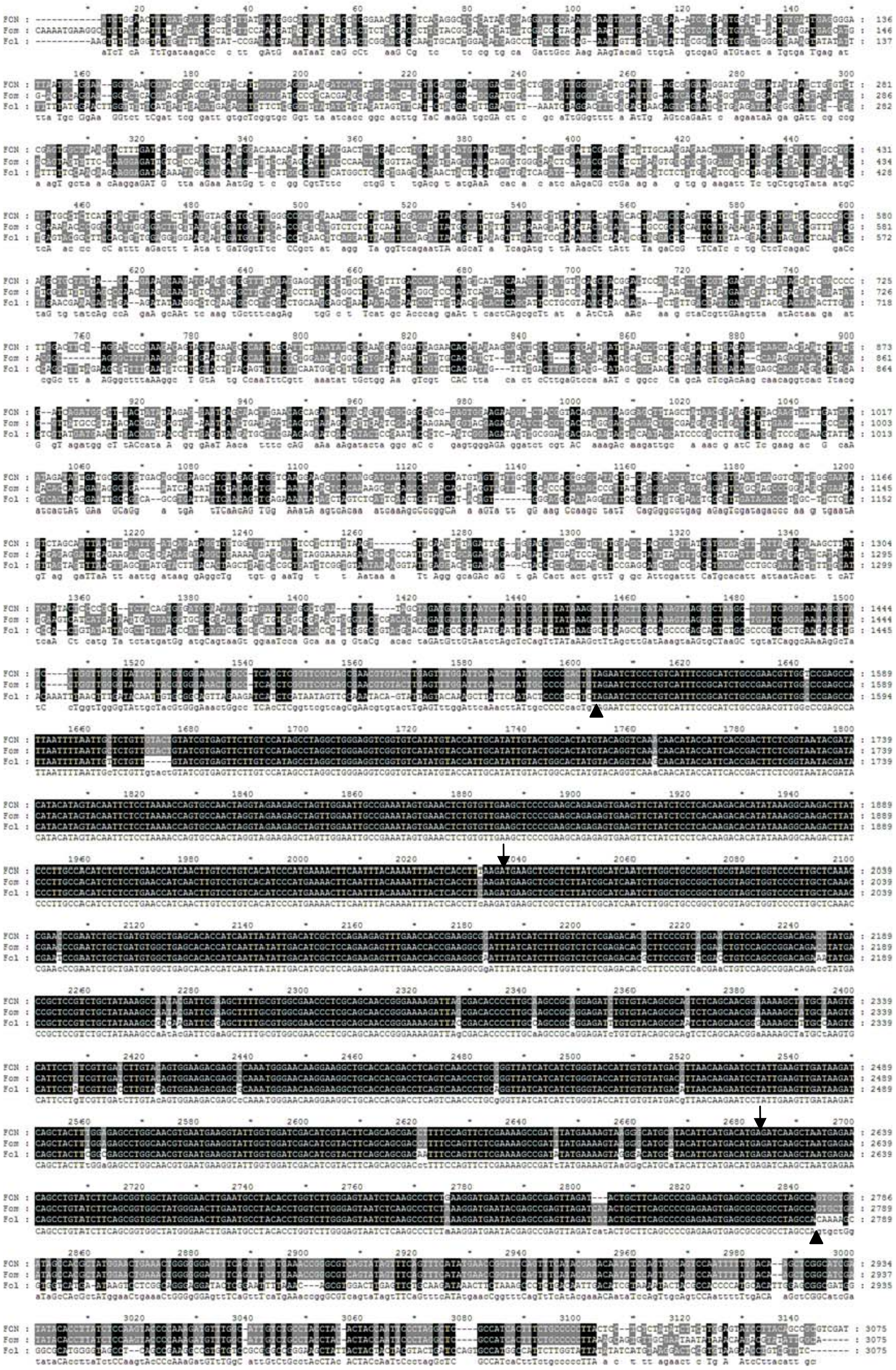

Supplementary Fig. S2 Sequence alignment of *SIX6* gene

Fon, *F. oxysporum* f. sp. *niveum*; Fom, *F. oxysporum* f. sp. *melonis*; Fol: *F. oxysporum* f. sp. *lycopersici*. The sequence of *FonSIX6* was cloned by Chromosome walking in this study. The sequences of *FolSIX6* and *FomSIX6* are derived from the genome sequences of Fol and Fom (http://www.broad.mit.edu). The arrow points to the start codon and stop codon; the black triangle is -434 position (directly adjacent to the start codon) and +157 position.

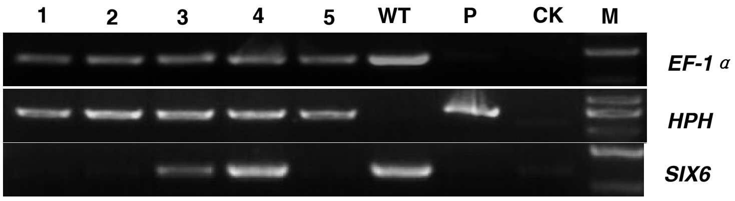

Supplementary Fig. S3 Confirmation of race1 knockout mutants

The transformants 1, 2, 5 were true mutants with *FonSIX6* gene deletions. lanes 1-5, transformants; WT, wild type race 1; P, *FonSIX6* knockout constructs; CK, ddH2O as a negative control; M, DNA maker; *EF-1α*, elongation factor 1*α* as positive controls; *HPH*, hygromycin resistance gene; *SIX6*, *FonSIX6* gene.

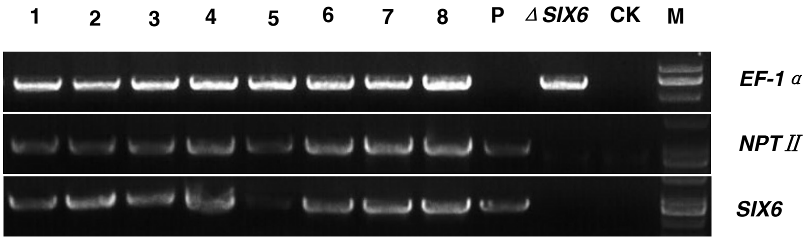

Supplementary Fig. S4 Confirmation of *ΔFon1SIX6* complementation mutants

The transformants were true mutants with *FonSIX6* gene except 5. lanes 1-8, transformants; P, *FonSIX6* complementation constructs; *ΔSIX6*, race 1 *FonSIX6* gene knockout mutants; CK, ddH2O as a negative control; M, DNA maker; *EF-1α*, elongation factor 1*α* as positive controls; *NPTⅡ*, neomycin phosphotransferase Ⅱ gene; *SIX6*, *FonSIX6* gene.

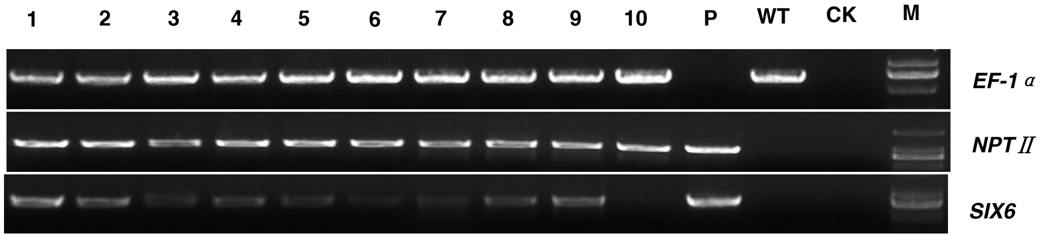

Supplementary Fig. S5 Confirmation of race 2 complementation mutants

The transformants were true mutants with *FonSIX6* gene except 10. lanes 1-10, transformants; P, *FonSIX6* complementation constructs; WT, race 2; CK, ddH2O as a negative control; M, DNA maker; *EF-1α*, elongation factor 1*α* as positive control; *NPTⅡ*, neomycin phosphotransferase Ⅱ gene; *SIX6*, *FonSIX6* gene.
